# Evaluating the underlying physiological and molecular mechanisms in the system of rice intensification performance with *Trichoderma*-rice plant symbiosis as a model system

**DOI:** 10.3389/fpls.2023.1214213

**Published:** 2023-08-24

**Authors:** Febri Doni, Ratu Safitri, Nurul Shamsinah Mohd Suhaimi, Mia Miranti, Nia Rossiana, Muhamad Shakirin Mispan, Azwir Anhar, Norman Uphoff

**Affiliations:** ^1^ Department of Biology, Faculty of Mathematics and Natural Sciences, Universitas Padjadjaran, Jatinangor, West Java, Indonesia; ^2^ Institute of Biological Sciences, Faculty of Science, University of Malaya, Kuala Lumpur, Malaysia; ^3^ Centre for Research in Biotechnology for Agriculture (CEBAR), University of Malaya, Kuala Lumpur, Malaysia; ^4^ Department of Biology, Faculty of Mathematics and Natural Sciences, Universitas Negeri Padang, Padang, West Sumatra, Indonesia; ^5^ Department of Global Development, Cornell University, Ithaca, NY, United States

**Keywords:** system of rice intensification, microbiomes, plant-microbe interactions, *Trichoderma*, gene modulation, rice

## Abstract

The system of rice intensification (SRI) is an extensively-researched and increasingly widely-utilized methodology for alleviating current constraints on rice production. Many studies have shown physiological and morphological improvements in rice plants induced by SRI management practices to be very similar to those that are associated with the presence of beneficial microbial endophytes in or around rice plants, especially their roots. With SRI methods, grain yields are increased by 25-100% compared to conventional methods, and the resulting plant phenotypes are better able to cope with biotic and abiotic stresses. SRI management practices have been shown to be associated with significant increases in the populations of certain microorganisms known to enhance soil health and plant growth, e.g., *Azospirillum, Trichoderma*, *Glomus*, and *Pseudomonas*. This article evaluates the effects of applying *Trichoderma* as a model microbe for assessing microbial growth-promotion, biological control activity, and modulation of gene expression under the conditions created by SRI practices. Information about the molecular changes and interactions associated with certain effects of SRI management suggests that these practices are enhancing rice plants’ expression of their genetic potentials. More systematic studies that assess the effects of SRI methods respectively and collectively, compared with standard rice production methods, are needed to develop a more encompassing understanding of how SRI modifications of crops’ growing environment elicit and contribute to more robust and more productive phenotypes of rice.

## 1 Introduction

The System of Rice Intensification (SRI), a climate-smart, yield-increasing methodology, has been validated in over 60 countries and is currently practiced by probably more than 20 million rice farmers (SRI-Rice, 2023). SRI practices create more favorable conditions for plant growth by (a) minimizing inter-plant competition, with seedlings planted singly, not in clumps, and with wide spacing between them, (b) starting with very young seedlings if establishing the crop by transplanting, taking care to minimize trauma to plant roots, while (c) creating healthier soil conditions, by (i) managing water so as to avoid anoxic soil conditions and also water-stress, (ii) active surface soil aeration during mechanical weeding, and (iii) increasing organic soil amendments (Thakur and Uphoff, 2017; Thakur et al., 2020; Mishra et al., 2021). Grain yields from SRI plants are generally 25-100% higher than from non-SRI rice crops (Nirmala et al., 2021; Shah et al., 2021; Thakur et al., 2022). In a number of ways, rice plants grown with SRI methods show superior morphological and physiological characteristics (Thakur et al., 2011; Thakur et al., 2023).

There is considerable evidence that using SRI’s methods results in improved rice phenotypes compared to rice plants of the same genotype that are grown with conventional methods (Thakur et al., 2010; Mishra and Salokhe, 2011; Hidayati et al., 2016). These differences have been reviewed previously articles, e.g., Thakur et al. (2016; 2023); Uphoff and Thakur (2019). Outcomes so observable and measurable must be associated in some way with biological processes and interactions that can be studied, even or especially at the molecular level. Relationships that have been documented by research should be clarified and accounted for through systematic scientific studies.

Under SRI management, paddy soils are maintained mostly under unflooded conditions, receiving additional oxygen through soil-aerating mechanical weeding that is conducive for the growth of aerobic soil microorganisms (Doni et al., 2022). Also, with SRI there is little or no reliance on synthetic fertilizers that can be toxic for some microbes and disruptive to their communities (Stoop et al., 2017). These changes in agronomic practices suggest that the observed improvements in the growth and yield of rice plants under SRI management might be understood in terms of interactions between plants and their microbiomes, understandable in terms of specific molecular mechanisms. Recent microbiological research has shown, for example, that aerobic soil conditions favor the development of beneficial soil organisms, from microbes to earthworms, while anaerobic conditions are more suitable for less beneficial and even adverse microorganisms (Thapa et al., 2018; Klinnawee et al., 2021).

SRI practices have also been observed to positively influence soil physicochemical properties such as increased nutrient cycling, improved soil structure, and enhanced water-holding capacity, resulting in better nutrient availability for plant growth (Thakur et al., 2016). Additionally, SRI practices can lead to a shift in microbial community composition, favoring beneficial genera like *Pseudomonas* and mycorrhizal fungi while suppressing pathogenic microorganisms (Stoop et al., 2017; Rokins et al., 2022). These alterations in the physicochemical and microbial properties of soil contribute to improved soil health, nutrient availability, and disease suppression, ultimately improving plants’ productivity (Doni et al., 2019a; Mattoo et al., 2023).

Research is just getting started on SRI effects on plant-microbiome interactions and associated molecular mechanisms. But several studies have indicated that SRI methodology affects the abundance and structure of microbial communities in the soil, on the plant, and within the plant, influencing symbiotic interactions between the rice plants and certain microorganisms (Anas et al., 2011; Uphoff et al., 2013; Doni et al., 2019a). For instance, *Trichoderma asperellum* SL2, an aerobic symbiotic fungus, has been employed as a model microbe to investigate its impact on the agronomic and molecular characteristics of rice plants under both SRI and conventional methods. The findings indicated that SRI provided favorable conditions for microbial growth which led to significant increases in rice growth, desirable physiological traits, yield, disease resistance, and gene expression. In contrast, conventional methods showed limitations in supporting the growth of *T. asperellum* SL2. These contrasting outcomes are reviewed in this article, summarizing previously published research (Doni et al., 2017; Doni et al., 2019b; Doni et al., 2023). So, we are considering relationships that are not just conjecture.

This paper (i) reports on the diversity and dynamics of soil microbial communities as influenced by using SRI methods, (ii) compares rice phenotypes under SRI vs. non-SRI management, both with and without inoculation with *T. asperellum* SL2, and then (iii) discusses molecular mechanisms that could be enhancing rice plant performance under SRI management with respect to plant-microbe interactions and the modulation of gene expression.

## 2 The diverse and dynamic structure of microbial communities under SRI management

The positive performance of SRI methods can be studied at the level of plant interactions with soil microbes and their association with plant organs. Some of these microbes are or become symbiotic endophytes that reside within the plants’ cells and organs. Interest in this aspect of SRI impact began with an early study in Madagascar which indicated that the effects of SRI practices were associated with an abundance of N-fixing bacteria (*Azospirillum*) living within the roots of rice plants (Andriankaja, 2001). Replicated trials showed that SRI management practices multiplied the populations of this microorganism which is known to be beneficial in plant roots by almost 20-fold, with positive effects on both plant tillering and yield (Uphoff and Randriamiharisoa, 2002).

Conducting large factorial trials on two different types of soil (clay vs. loam), this study evaluated the effects of this diazotrophic bacteria in response to SRI management practices, respectively and collectively, compared to conventional rice cultivation methods. Under SRI management, there was a huge increase in the populations of root-endophytic *Azospirillum*, greater not surprisingly in clay soil compared to loam soil, as seen in **Table 1**. The colonization by *Azospirillum* within rice plant roots was associated with significantly more tillers per plant and with significant enhancement of crop yield (Andriankaja, 2001; Randriamiharisoa et al., 2006). The colonization of rice plant roots by this symbiotic microbe was associated with SRI-grown plants having 4x more tillers and almost 6x greater crop yield.

**Table 1:** Symbiotic microbe *Azospirillum* populations in rice plant roots associated with cultivation practices (SRI vs. conventional) and nutrient amendments on different soil types in Madagascar.

	*Azospirillum* count in roots (10^3^ ml^-1^)	Tillers plant^-1^	Yield (t ha^-1^)
Clay Soil			
Conventional rice cultivation with no nutrient amendments	65	17	1.8
SRI practices with no soil nutrient amendments	1,100	45	6.1
SRI practices with amendments of inorganic fertilizer (NPK)	450	68	9.0
SRI practices with amendments of compost	1,400	78	10.5
Loam Soil			
SRI practices with no soil nutrient amendments	75	32	2.1
SRI practices with amendments of compost	2,000	47	6.6

Source: Randriamiharisoa (2002); Randriamiharisoa et al. (2006).

In a later study done in China (Lin et al., 2011), the largest population numbers of actinomycetes, a plant-beneficial soil microbe, were observed in the soil around rice plant roots under SRI cultivation with fully organic fertilization. In SRI test plots that were alternately wetted and dried, the number of actinomycetes increased by 292% when the soil received 100% organic fertilization, compared to the populations in soil that received only 25% of its nitrogen amendments from an organic source, with the other 75% coming from inorganic fertilizer. All of the test plots were amended with the same amount of N, so the amount of this nutrient amendment available was not a variable – only its composition, varying the proportion of N that was provided from organic and from inorganic sources.

In the test plots under conventional management with continuous flooding, raising the share of N soil amendment from 25% organic to 100% organic enhanced the number of actinomycetes by 78%, by only about one-quarter as much as with SRI management and unflooded soil. Populations of actinomycetes were thus seen to respond quite differently to SRI vs. conventional irrigation and to organic vs. inorganic sources for soil nutrition (Lin et al., 2011).

In trials conducted in northern Thailand, the community structures of bacteria and archaea showed significant changes under SRI practice as compared with what was found in conventionally-managed rice fields. Community compositions of these soil microbes started to change during the vegetative phase of rice growth, just after the practice of alternating wetting and drying of SRI plots began; and the microbial communities continued to diverge between the two respective strategies until the water management for both systems was made the same again just before harvesting (Sooksa-Nguan et al., 2010). This indicated that bacterial and archaeal communities were responding quite differently to the regime for water management.

With regard to fungi, another study in Thailand found by metagenomic sequencing of roots’ rhizospheres that under SRI management there were two genera of arbuscular mycorrhizal fungi (AMF) present, colonizing rice plant roots, *Glomus* and *Acaulospora*, whereas with conventional cultivation, only one genus (*Glomu*s) could be detected in the roots of rice plants (Watanarojanaporn et al., 2013). These communities of fungi establish symbiotic associations with the roots of most terrestrial plants and thereby enhance plants’ nutrient uptake by extending the volume of soil from which nutrients can be accessed. They also assist plants in coping with biotic and abiotic stresses (Diagne et al., 2020). But to function, these beneficial fungi need aerobic soil conditions, which SRI methods provide them, whereas conventional rice-growing practices do not.

In another study, this one done in India, fluorescent pseudomonads, plant‐beneficial bacteria that reside in the rhizosphere around plant roots, were found in abundance in an SRI field compared to a non-SRI field (Suresh et al., 2014). These bacteria are well known to promote plant growth and to induce host-plant resistance toward biotic and abiotic stresses.

A recent study in India employing 16S rRNA Illumina amplicon sequencing compared the bacterial communities in the rhizosphere soil under different systems for rice production, i.e., conventional, aerobic, and SRI. The results showed that the values of the alpha diversity index for soil bacteria were higher with SRI management compared to both conventional and aerobic cultivation methods. Higher values of the alpha diversity index indicated that there was more relative abundance and/or a greater number of bacterial species. The results of this study also showed that *Pseudomonas* and *Clostridium* were found to be the most dominant genera in SRI plots compared to other plots (Rokins et al., 2022). These two genera of bacteria are well known to be involved in the promotion of plant growth and in the alteration of plants’ genetic potentials.

Soil microbial activity also plays a critical role in enhancing the availability of macro- and micronutrients within the rice rhizosphere (Anas et al., 2011; Shahane et al., 2019). For example, cyanobacteria-based biofilm inoculants were found to be more effective under SRI crop management than under conventional rice management for increasing the concentrations of zinc, copper, iron, and manganese measurable in the rice grain. Also, there was a significant increase in the activity of defense-related and pathogenesis-related enzymes, as well as in the yield parameters of rice plants (Adak et al., 2016; Shivay et al., 2022).

Aerobic and organic soil conditions under SRI also favor greater root growth and increased nutrient acquisition by organically-fertilized rice plants, with reduced accumulation of toxic elements in the soil such as Fe^2+^ and Mn^2+^ (Turmel et al., 2011). Research reported by Anas et al. (2011) showed that SRI management, in addition to enhancing the populations of soil microorganisms, increases enzymatic activity in the rhizosphere and the availability there of nutrients such as phosphorus, nitrogen, and carbon, which are essential for the growth of both plants and soil organisms.

A review of the literature on microbial populations associated with SRI vs. non-SRI cultivation methods indicates that with SRI methods, soil biological activities are increased, and the numbers and diversity of beneficial microorganisms are enhanced (Doni et al., 2019a). These results are summarized in **Table 2**.

**Table 2:** Increases in microbial populations under SRI vs. conventional crop managements.

Increase in	SRI > Conv.
Total bacteria (log_10_ g^-1^ dry soil)	3%
Total actinomycetes (log_10_ g^-1^ dry soil)	6%
Microbial biomass carbon (mg kg^-1^ soil)	14%
Total microbes (CFUs g^-1^ soil)	65%
N_2_-fixers (log_10_ g^-1^ dry soil)	6%
Phosphate-solubilizing microbes (CFUs g^-1^ soil)	79%
Microbial biomass nitrogen (mg kg^-1^ soil)	61%
*Azospirillum* (10^5^ ml^-1^ root)	1,592%
*Azotobacter* (CFUs g^-1^ soil)	95%
Total diazotroph population (g^-1^ dry soil)	117%
Phosphobacteria (g^-1^ dry soil)	35%
Proteobacteria^*^	10%
Alpha diversity index (Chao1)^*^	4%
Alpha diversity index (Fisher)^*^	2%

Source: Summarized from Doni et al. (2019a) and Rokins et al. (2022). Percentages are rounded off.

^*^Based on 16S rRNA metagenomic analysis.

## 3 Enhanced rice performance under SRI management with the inoculation of an endophytic microbe: *Trichoderma asperellum* SL2

Several studies have been performed in recent years to elucidate the influence of SRI methods on symbiotic interactions between rice plants and their associated microbes. The influence of SRI methods on microbial plant-growth promotion capacity has been investigated under gnotobiotic conditions using the symbiotic, plant-beneficial fungus, *T. asperellum* SL2 (**Table 3**).

**Table 3:** Agronomic and physiological enhancement in rice plants with *T. asperellum* SL2 inoculation under SRI management system.

Plants’ growth stages	Agronomic advantages	Physiological advantages
Seedling stage	• More vigorous seedlings • Better growth of shoots and roots	• Higher leaf chlorophyll content
Vegetative stage	• More tillers • More number of leaves • Better growth of shoots and roots	• Higher leaf chlorophyll content • Higher nutrient uptake • Higher photosynthetic rate • More stomatal number • Higher stomatal conductance • Lower transpiration rate • Higher water use efficiency
Ripening stage	• More panicles • Better growth of shoots and roots	• Higher leaf chlorophyll content • Higher photosynthetic rate
Harvest stage	• More grains per panicle • Higher % filled grains • Heavier grains	

Source: Summarized from Doni et al. (2017).

The trials showed that combining SRI methods with *T. asperellum* SL2 inoculation significantly increased the growth and yield of rice plants as well as their nutrient uptake, rate of photosynthesis, stomatal conductance, chlorophyll content, and numbers of stomata, compared to both (a) plants grown under SRI methods without *T. asperellum* inoculation, and (b) plants grown conventionally and inoculated with *T. asperellum* (Doni et al., 2017).

When this relationship was assessed under field conditions, results indicated that there was yield enhancement by more than 30% with *T. asperellum* SL2 inoculation and SRI management compared to SRI management alone (Doni et al., 2018a). The measured changes and increases in rice plants’ morphological and physiological characteristics with *T. asperellum* SL2 inoculation were the same as had been reported from previous research evaluating the effects of SRI methods by themselves; SRI-grown plants were shown to have superior agronomic and physiological performance, specifically, deeper and better distributed root systems, higher rates of photosynthesis, higher chlorophyll content in their leaves, and higher yield (Thakur et al., 2010).

Parallel research has been conducted assessing possible synergistic relationships between SRI and *T. asperellum* SL2 for their joint effect in controlling the major rice disease of sheath blight caused by the pathogenic fungus *Rhizoctonia solani*. These results, discussed at more length in Doni et al. (2023), showed that plants inoculated with *T. asperellum* SL2 and grown under SRI conditions had the lowest length of sheath-blight lesions, lower total length of lesions, a lower susceptibility index for sheath blight, with lower disease extent and total scoring scale compared to plants that had been grown either (a) under SRI management without *T. asperellum* SL2 inoculation, or (b) with conventional methods whether with or without this inoculation. The data indicated that the combined effects of *T. asperellum* SL2 inoculation and SRI methods synergistically boosted the rice plants’ resistance toward sheath blight disease (Doni et al., 2023). This was not a novel finding in that in both Vietnam and India, SRI-grown plants have been found to be more resistant to sheath blight as well as to other pests and diseases (Dung, 2007; Chintalapati et al., 2023).

**Figure 1:**
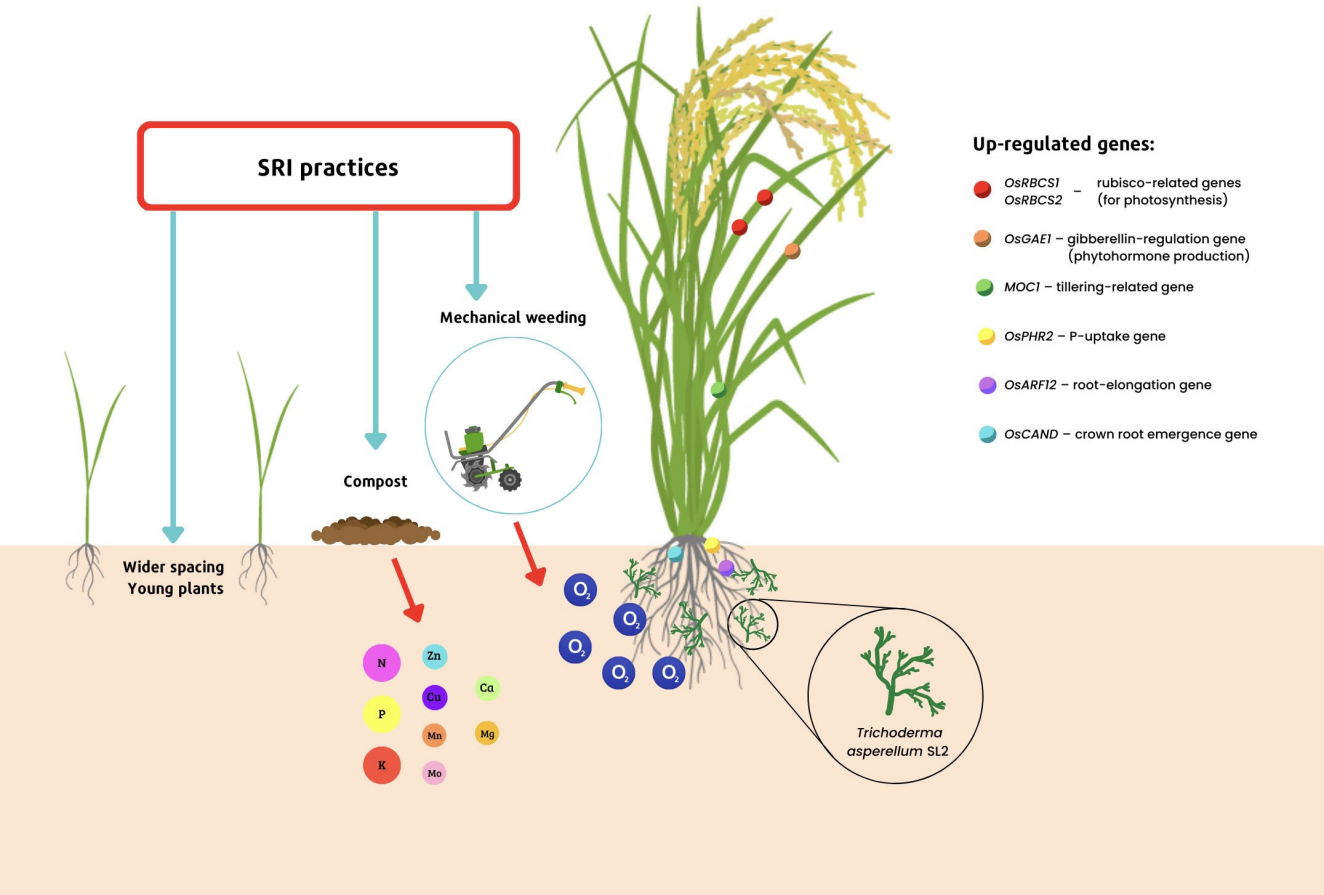
Transcriptomic profiling of *Trichoderma*-rice plant interactions under SRI management.

Further examination of rice plants’ gene expression using transcriptomic analysis has shown that rice plants inoculated with *T. asperellum* SL2 and grown with SRI methods have significantly greater expression of their genes that relate to the synthesis of crucial enzymes which are involved in the process of photosynthesis, including genes supporting the synthesis of Rubisco (*RBCS*, *OsRBCS1*, and *OsRBCS2*). Other genes that were significantly up-regulated in *Trichoderma*-inoculated, SRI-grown plants included two genes involved in stress tolerance (*CYP38* and *CYP20-2*); one gene regulating the synthesis of the critical phytohormone gibberellin (*OsGAE1*); a gene for tillering (*MOC1*); one regulating the uptake of phosphorus (*OsPHR2*); another related to root elongation (*OsARF12*); and a gene that controls crown root emergence (*OsCAND*) – compared to rice plants that had been inoculated with *Trichoderma* but grown conventionally, and to SRI-grown plants that had no *Trichoderma* inoculation (Doni et al., 2018b; Doni et al., 2019b). These effects are shown in **Figure 1**. The heightened gene expression identified by transcriptomic analysis was consistent with the phenotypic changes that had been previously reported by Thakur et al. (2010) in SRI-grown rice plants.

Further research in controlled trials has shown that the enhanced soil microbial abundance and activity promoted by the application of microbial-based biofertilizer or certain microbial-based inoculants like *T. asperellum* SL2 are associated not only with better rice plant performance compared to that of plants that have no inoculation, but also with desirable changes in the rice plants’ gene expression which would account for the improvements in phenotype (Doni et al., 2018a; Doni et al., 2018b; Doni et al., 2019b).

There are many similarities between the changes in rice plant phenotypes that are observed, respectively, with (a) the application of SRI practices and (b) with inoculation with *T. asperellum* SL2, as seen in **Table 4**. It is understood that correlation does not establish causation, but the extent of correspondence does suggest that more is involved here than coincidence.

**Table 4:** Similarities between the effects of SRI and *T. asperellum* SL2 inoculation on rice plants’ morphology and physiology.

Agronomic, physiological, and molecular traits	SRI	*T. asperellum* SL2
Better shoot and root growth	+	+
More number of tillers	+	+
More number of leaves	+	+
More number of panicles	+	+
More grains per panicle	+	+
Higher leaf chlorophyll content	+	+
Higher nutrient uptake	+	+
Higher photosynthetic rate	+	+
Higher stomatal conductance	+	+
Higher stomatal numbers	+	+
Lower transpiration rate	+	+
Better water use efficiency	+	+
Better resistance to disease	+	+
Better resistance to biotic stresses	+	+
Large-scale up-regulation of genes related to energy metabolism, plant defense response, plant response to abiotic stimuli, modulation of reactive oxygen species (ROS), and root morphogenesis	NM	+

+, Observed; NM, not measured.

Further studies should assess the effects of SRI practices on rice plants’ gene expression.

Source: Summarized from Doni et al. (2017; 2018b; 2019b) and Thakur et al. (2010; 2018).

Little research thus far has directly assessed SRI management practices as an independent variable having demonstrable effects on plants’ molecular signaling and gene expression, however. And we are not suggesting here that SRI effects are attributable only to microbial activity. Wider spacing of plants reduces shading and contributes to increased light interception even with much-reduced plant density. It affects rice plants’ physical architecture, with tillers more horizonal and leaves more vertical. And stopping continuous flooding of rice paddies avoids necrosis and stunting of roots due to hypoxia. SRI is difficult to evaluate because its multiple effects, and any associated synergy among them, complicate both research design and analysis. But there is reason to conclude that more systematic and in-depth studies at the molecular level should be undertaken to assess the effects of SRI practices in terms of particular cellular and molecular processes affected, which we consider next.

## 4 Possible molecular mechanisms for enhanced rice plant phenotypes under SRI management

Plants’ microbiomes play significant roles in their nutrient acquisition and mobilization as well as in conferring protection against various environmental stresses (Harman et al., 2021; Zhang et al., 2021; Trivedi et al., 2022). Plant growth enhancement by interaction between plants and their associated soil microbiomes can be seen by looking at morphological and physiological differentials, but these effects must have underlying molecular processes and some regulation of gene expression (Ge et al., 2023). Plant-soil microbiome interactions are part of complex biological systems in which there is what is referred to as ‘genetic cross-talk’ associated with the altered expression of specific genes (Vaishnav and Choudhary, 2019; Abdullah et al., 2021).

When rice plants respond to stress morphologically or physiologically, certain sets of genes are being up-regulated (or down-regulated) in the stressed rice plants. Such reactions could be triggered by having been inoculated with symbiotic microbes. For example, the inoculation of rice plants with *Bacillus amyloliquefaciens* under saline soil conditions has been found to increase the inoculated plants’ growth and salt-tolerance as there is modulated expression of at least 14 identifiable genes related to the plants’ defense mechanisms (Nautiyal et al., 2013).

Rice plants treated with *T. harzianum* have been shown to exhibit greater drought-tolerance as compared with untreated control plants (Shukla et al., 2012; Pandey et al., 2016). There were significant changes in a number of mechanisms that can protect the plants from drought stress: synthesis of malondialdehyde and proline, higher superoxide dismutase level, greater plant height, more total dry matter, relatively greater chlorophyll content, leaf rolling, less leaf-tip burn, and fewer scorched leaves, all associated with inoculation with *T. harzianum*. This particular study demonstrated also an up-regulation in the expression of genes for synthesizing aquaporin and dehydrin genes in the *T. harzianum*-treated rice plants (Pandey et al., 2016).

More recently, *T. asperellum* has been reported to be a potential biocontrol agent that can suppress rice blast disease caused by *Magnaporthe oryzae*. This study showed that inoculation with *T. asperellum* enhanced several important physiological traits – total chlorophyll content, antioxidant enzymes, and water use efficiency – as well as the expression of certain defense-related genes such as *J10sPR10* and *LOX-RLL* (Sousa et al., 2020).

The diazotrophic bacteria *Sinorhizobium meliloti* 1021, which with leguminous plants is a root-nodule endosymbiont, can also form endophytic associations with rice, a plant that is not a legume. After *S. meliloti* 1021 colonizes the roots of rice plants, it migrates upwards and colonizes aboveground plant tissues and organs, such as the stem base, leaf sheath, and leaves where the bacteria can develop high populations (Chi et al., 2005). *In situ* imaging analysis has shown that local endophytic population densities of this microorganism can reach as high as 9 × 10^10^ rhizobia per cm^3^ in the inhabited host plant tissues. The symbiotic interaction of microbes and plant is correlated with the enhancement of root and shoot biomass, photosynthetic rate, stomatal conductance, transpiration velocity, water utilization efficiency, and flag leaf area.

Follow-up proteomic analysis has demonstrated that after rice plants have been inoculated with *S. meliloti* 1021, proteins involved in nine different functional categories are up-regulated (or down-regulated), meaning that significantly more (or fewer) proteins are synthesized inoculated plants than in uninoculated plants. Proteins related to photosynthesis are up-regulated in the leaf sheaths and leaves of inoculated rice plants, while proteins involved in defense mechanisms are up-regulated particularly in the roots (Chi et al., 2010).

Further research has shown that the molecular mechanisms affected by interactions between the rice plants and *S. meliloti* 1021 produce certain bioactive signals emitted by *S. meliloti* 1021 during the early stages of interaction that are recognized by receptor proteins on the rice root cells. Upon the rice plant’s recognition and transduction of these signals from *S. meliloti* 1021, many differentially-expressed genes (DEGs) are induced in the shoots of the rice plant, ones that are essential for phytohormone production, photosynthetic efficiency, carbohydrate metabolism, cell division, and cell wall expansion.

This recognition and transduction modulate cell-cycle regulator genes that lead to accelerated cell division. The plant-microbe interactions also enhance other processes that culminate in greater plant growth resulting from an intensification of physiological processes like energy metabolism, phytohormone production, and cell wall expansion (Wu et al., 2018). As rice is often taken as a model of monocotyledon performance as well as being a globally important cereal crop, molecular interactions between rice plants and microorganisms have been fairly widely studied, as seen in the many research findings summarized in **Table 5**.

**Table 5:** Examples of reported microbially-mediated gene expression in rice plants.

Microbes	Key results	References
*Glomus mosseae*	Expression of a lipid transfer protein gene was up-regulated in rice roots in response to colonization of *G. mosseae.*	Blilou et al. (2000)
*G. intraradices*	More than 200 genes were modulated upon inoculation of rice roots with *G. intraradices*. Some of the genes that responded to *G. intraradices* colonization were involved in the uptake of phosphate.	Guimil et al. (2005)
*Azospirillum* spp.	A large set of genes was significantly up-regulated in rice roots after inoculation with *Azospirillum*. The DEGs affected by inoculation are ones involved in primary metabolism, transport, regulation of transcription, and protein fate.	Drogue et al. (2014)
Arbuscular mycorrhizal fungi (AMF)	AMF-enhanced gene expression in the crown roots of rice plants altered secondary cell wall synthesis and reprogrammed crown root characteristics.	Gutjahr et al. (2015)
*Pseudomonas fluorescens*	*P. fluorescens* was found to regulate antioxidative reactions and induce defense-related genes that are involved in drought-stress alleviation.	Singh et al. (2020a)
Co-inoculation of *Trichoderma asperellum* and *P. fluorescens*	Co-inoculation of rice roots significantly up-regulated *PAL*, *cCuZn-SOD* and *CAT* genes, leading to better growth and development and to tolerance of abiotic stresses.	Singh et al. (2020b)
*Stenotrophomonas* spp. and *Microbacterium oleivorans*	60% reduction in the severity of rice blast disease caused by *M. oryzae* on rice leaves; inoculation triggered expression of plant-defense genes such as *OsCEBiP*, *OsCERK1*, *OsEDS1*, and *OsPAD4*.	Sahu et al. (2021)
*Streptomyces hygroscopicus*	Induced mitigation of Fe deficiency in rice plants occurred through modulation of the expression of key genes involved in the chelation, solubilization, reduction, and translocation of iron.	Cao et al. (2021)
*Phomopsis liquidambaris*	*P. liquidambaris* significantly up-regulated nutrient-transporter genes as well as improved the quality and mineral nutrition of rice grain by optimizing nutrient assimilation and partitioning.	Tang et al. (2022)

## 5 Conclusions

SRI practices for growing irrigated rice have been seen repeatedly to result in improved morphology of rice plants and in physiological processes that result in better growth and higher grain production. However, these assessments have been conducted without reference to or examination of the molecular processes that were occurring inside the rice plants’ cells. Observable effects on rice plants’ growth and performance must have some causal processes that are occurring concomitantly at the cellular, molecular, and genetic levels.

The beneficial interactions that take place between rice plants and their associated microbiomes are becoming of ever-greater interest. It is clear that beneficial microbes play a pivotal role in the functioning of rice plants by influencing physiological and molecular processes. Transcriptome and proteome profiling of rice plants following their inoculation with certain microbes has identified specific genes that are involved in key physiological and molecular processes in rice plants being up-regulated or down-regulated compared to uninoculated control plants. This was evidently in response to the inoculation because other factors were being controlled.

These changes result in greater or lesser synthesis of certain proteins, including enzymes, within plant cells. This would differ perhaps in kind but not in degree from the effects of certain endophytic microorganisms that are already resident in plants, these microbes being affected by management practices that determine the environment of plants (and microbes). It is unlikely to be just coincidence that the effects of SRI management practices and of microbial inoculation are so similar.

The finding that the accelerated/improved performance of rice plants being grown with SRI management correlates strongly with the effects of inoculating rice plants with certain endophytic microbes like *Trichoderma* suggests that SRI methodology may itself be affecting the microbial populations in, on, and around rice plants, and that these communities in turn affect their hosts’ growth and physiological characteristics, as well as the molecular processes that underlie plant growth and development as well as the plants’ sickness and health. The results of rice plant inoculation have corresponded very closely to the effects observed with SRI practices, so it is anticipated that these practices are inducing similar dynamics and effects at the molecular level that are mediated by the plant’s microbiome.

However, much research remains to be carried out, to elucidate the effects of SRI management at the molecular level, compared with such effects from non-SRI practices. It will be interesting for future studies to analyze the patterns of gene expression and protein synthesis in rice plants grown under SRI management vs. conventional practices through comprehensive use of transcriptomic and proteomic analysis. Such a study has been initiated in Indonesia by researchers from Meiji University in Japan, and their findings should advance our understanding of what is going on at the molecular level in SRI-grown rice plants. Multi-omics approaches can provide a systems-level view of the complex interactions and regulatory networks that occur in plant growth and performance, helping to identify key biological processes and pathways associated with SRI-related crop improvements.

## Author contributions

Conceptualization, FD. writing—original draft preparation, FD, NU, MM, NS, and MSM. writing—review and editing, FD, NU, MM, NS, MSM, RS, NR, and AA. visualization, FD, and AA. funding acquisition, FD. All authors contributed to the article and approved the submitted version.
